# Metabolomics and lipidomics signature in celiac disease: a narrative review

**DOI:** 10.1007/s10238-024-01295-2

**Published:** 2024-02-10

**Authors:** Mohammad Rostami-Nejad, Nastaran Asri, Sajjad Bakhtiari, Ensieh Khalkhal, Sepehr Maleki, Mostafa Rezaei-Tavirani, Somayeh Jahani-Sherafat, Kamran Rostami

**Affiliations:** 1 Celiac Disease and Gluten Related Disorders Research Center, Research Institute for Gastroenterology and Liver Diseases, Shahid Beheshti University of Medical Sciences, Tehran, Iran; 2 Gastroenterology and Liver Diseases Research Center, Research Institute for Gastroenterology and Liver Diseases, Shahid Beheshti University of Medical Sciences, Tehran, Iran; 3 Proteomics Research Center, Faculty of Paramedical Sciences, Shahid Beheshti University of Medical Sciences, Tehran, Iran; 4 Department of Computer Science, University of Tabriz, Tabriz, Iran; 5 Laser Application in Medical Sciences Research Center, Shahid Beheshti University of Medical Sciences, Tehran, Iran; 6 Department of Gastroenterology, MidCentral DHB, Palmerston North, 4442 New Zealand

**Keywords:** Celiac disease, Metabolomics, Lipidomics, Biomarker

## Abstract

Celiac disease (CD) is a chronic immune-mediated inflammatory disease of the small intestine caused by aberrant immune responses to consumed gluten proteins. CD is diagnosed by a combination of the patients reported symptoms, serologic and endoscopic biopsy evaluation of the small intestine; and adherence to a strict gluten-free diet (GFD) is considered the only available therapeutic approach for this disorder. Novel approaches need to be considered for finding new biomarkers to help this disorder diagnosis and finding a new alternative therapeutic method for this group of patients. Metabolomics and lipidomics are powerful tools to provide highly accurate and sensitive biomarkers. Previous studies indicated a metabolic fingerprint for CD deriving from alterations in gut microflora or intestinal permeability, malabsorption, and energy metabolism. Moreover, since CD is characterized by increased intestinal permeability and due to the importance of membrane lipid components in controlling barrier integrity, conducting lipidomics studies in this disorder is of great importance. In the current study, we tried to provide a critical overview of metabolomic and lipidomic changes in CD.

## Introduction

Celiac disease (CD), first described in 1887, is a chronic immune-mediated inflammatory disease of the small intestine caused by intolerance to gluten proteins [1–3]. CD is known as one of the most common genetic disorders, with a reported global prevalence of 0.5–1% in the general population and its prevalence continues to rise [4–6]. The genetic susceptibility of CD involves human leukocyte antigen (HLA)-DQ2 and HLA-DQ8 heterodimers [7, 8]. Since HLA-DQ2 and HLA-DQ8 heterodimers explain almost 40% of the disease heritability, HLA typing should not be applied in diagnosis, but exclusively to clarify uncertain diagnoses, showing the low negative predictive value for CD diagnosis through HLA genotyping [9, 10]. Being rich in proline (Pro) and glutamine (Gln), gluten proteins are resistant to fully degrade by gastrointestinal tract enzymes, producing immunogenic gliadin peptides inducing inappropriate T-cell-mediated intestinal mucosal damages characterized by villous flattening, crypt hyperplasia, and intraepithelial lymphocytosis [11–13]. The disease can typically present with a broad spectrum of gastrointestinal and extra-intestinal symptoms and like many autoimmune diseases, CD might be associated with other autoimmune disorders like type 1 diabetes and autoimmune thyroiditis [14–18]. CD is diagnosed using a combined analysis of patients’ symptoms, a positive serologic test together with an endoscopic biopsy evaluation of the small intestine [19]. As about 3–5% of CD patients have negative serology results, intestinal architectural distortions are not exclusively related to CD pathogenesis, and the invasiveness of the intestinal biopsy evaluation method, novel approaches like new biomarkers need to be considered for accurate diagnosis of CD [20]. Moreover, adherence to a lifelong gluten-free diet (GFD) is considered the only available therapeutic approach for this disorder, which is not a conclusive therapy and researchers are looking for finding a new alternative method [21]. Metabolomic and lipidomic approaches are known as powerful tools to provide highly accurate and sensitive biomarkers, which are useful for predicting diagnosis, prognosis, and treatment responses [22–24]. Thanks to major advancements in analytical instruments and bioinformatic analysis platforms, over the past two decades, metabolomics and lipidomics have undergone significant advances, and their application in a wide range of research fields including health and diseases has attracted the attention of the healthcare system [25, 26]. The current study was aimed at providing a critical overview of available data about metabolomic and lipidomic changes in celiac disease, which may lead us to find a promising avenue to improve our understanding of this complex disorder-related biological biomarkers.

## Methods

### Search strategy

We conducted a review of metabolomics and lipidomics studies on CD and searched the electronic databases of PubMed, EMBASE, Scopus, and Web of Science for finding relevant studies published up to March 2023, with the following search terms: (“metabolomics” OR “metabonomics” OR “metabolome” OR “metabolic profiling” OR “metabolism”), AND (“lipidomes” OR “lipidomic” OR “lipidomics” OR “lipids”) AND (“celiac” OR “coeliac” OR “celiac disease” OR “CD” OR “CeD” OR “gluten enteropathy” OR “Gluten-Sensitive Enteropathy” OR “Nontropical Sprue” OR “Celiac Sprue”). Additional articles were identified through searching the reference lists from included studies. After collecting articles, further identification was performed based on inclusion and exclusion criteria.

### Inclusion and exclusion criteria

All English-language studies that evaluated metabolomics and lipidomics profiles of CD patients were considered. Studies were excluded if they were drug therapy response reports, animal studies, or in vivo studies.

## Results

### Metabolomic changes in CD

**Fig. 1:**
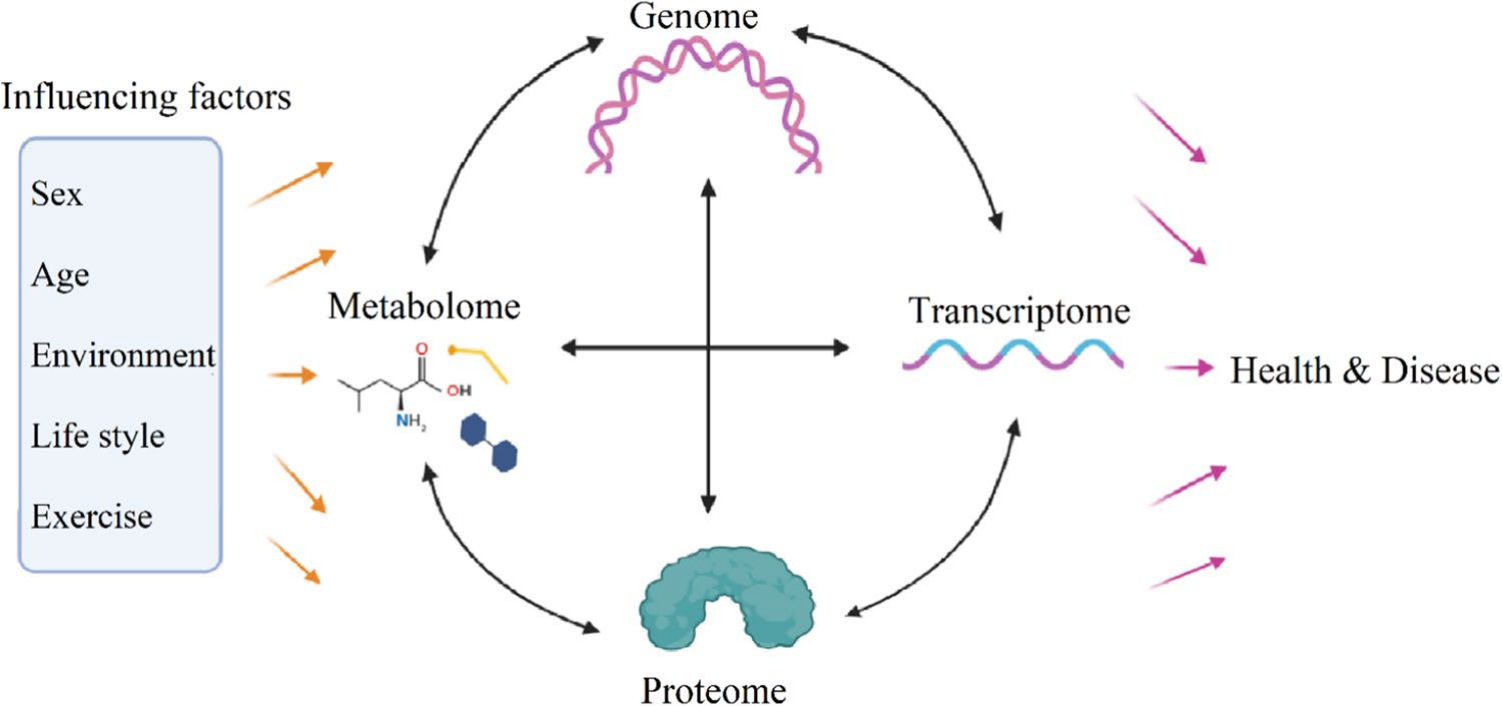
Interrelations between biological layers

Metabolomic is an analytical profiling technique emerging with the purpose of a comprehensive analysis of metabolites, which are downstream of the genome, transcriptome and proteome in biological specimens [27, 28]. Changes in gene expression levels have large effects on metabolic pathways and metabolites concentrations; and metabolites can also influence gene transcription [3, 29]. Actually, there are complex interrelations between the genome, transcriptome, proteome and metabolome; and sex, age, environment, lifestyle and exercise can affect all layers of the biological system (Fig. 1) [30].

Metabolomic is a more comprehensive, faster, and cheaper counterpart of clinical chemistry, that uses high throughput techniques to characterize small molecules in different biological fluids that are collected in non-invasive or minimally invasive ways like the serum, plasma, urine, saliva, seminal fluid and fecal extracts [31–34]. These approaches measure a large number of small molecules that are produced by specific cellular processes in response to physiological stimuli or in disease states and due to the genetic modifications, that provide a “snapshots” of the state of a cell and clearly express the complexity of disease [35–38]. Targeted and non-targeted metabolomics are two main categories of metabolomics analysis [39]. Targeted metabolomics encompasses the quantitative measurement of the known metabolites related to specific metabolic pathways [35, 40]. In contrast, non-targeted metabolomics analyses are based on the global identification of both known and unknown metabolites and identify as many metabolites as possible and provide more information than targeted metabolomics [40, 41]. These two approaches are used for measuring hundreds to thousands of metabolites [3]. Metabolomics expand current limited knowledge about diseases and provide significant information about various pathologies, such as cardiovascular diseases, neurological disorders, cancers, and celiac disease [42–47].

A clear difference was observed between metabolomic profiles of CD patients and healthy controls (HCs) in previously published reports, which indicated a metabolic fingerprint for CD deriving from alterations in gut microflora or intestinal permeability, malabsorption, and energy metabolism [3, 21].

#### Gut microflora and metabolite profile changes in CD

The human gastrointestinal tract is inhabited by thousands of bacterial species, termed the microbiota [48]. Microorganisms can undergo catabolism or anabolism to metabolize various compounds. Additionally, these metabolites can exert stimulation or inhibition effects on microbial growth [49]. Actually, the gut microbiota has regulatory effects on a variety of important metabolic functions [20]. The Celiac Disease Genomic, Environmental, Microbiome and Metabolomic (CDGEMM) study is a long-term research project focusing on predicting the onset of celiac disease in at-risk children. It involves collecting longitudinal blood and stool samples, along with questionnaires, from participants over a five-year period. Initial findings show the presence of specific microbial strains and metabolites associated with autoimmune and inflammatory conditions before the development of celiac disease, while other beneficial components decrease [50]. Another study on 102 children in the CDGEMM study from 2014–2022 aimed to investigate whether there are changes in intestinal permeability before the onset of celiac disease autoimmunity (CDA) in at-risk children. The research found that children who developed CDA experienced a significant increase in zonulin levels, a marker of gut permeability, in the months leading up to CDA diagnosis. Additionally, a higher number of antibiotic courses was associated with increased zonulin levels and potentially increased risk of CDA. These findings suggest that zonulin could be used as a biomarker for preclinical screening of celiac disease, and caution is advised regarding the use of multiple antibiotic courses in at-risk children [51]. Longitudinal analyses revealed increased abundance of certain species/strains/pathways/metabolites before CD onset, previously associated with autoimmune and inflammatory conditions, while others decreased before CD onset and have anti-inflammatory effects [52]. Koenig et al. [53] in a 2.5-year case study on sixty fecal samples of a healthy infant observed a gradual change in microbial community diversity. In more detail, before the introduction of solid food to the infant diet, the earliest microbiome was compromised of genes facilitating lactate utilization, along with the presence of plant polysaccharide metabolism-related genes, which made them metabolically ready to receive plant-derived foods. It was followed by a sustained increase in the abundance of *Bacteroidetes*, increased levels of fecal short-chain fatty acids, and carbohydrate utilization, vitamin biosynthesis, and xenobiotic degradation-related genes [53].

According to the reports, both CD and GFD can affect gut microbiota composition and bacterial activity through different mechanisms and with different etiologies [54]. Recent reports also refer to the possible role of intestinal microbiota in CD development [55–59]. The changes in CD patients’ microbiota are mainly accompanied by the increase in Gram-negative bacteria and the decrease in Gram-positive bacteria [60]. Leonard et al. [61] reported that cesarean section delivery was along with reduced levels of *Bacteroides vulgatus* and *Bacteroides dorei* and decreased folate biosynthesis pathway and higher levels of hydroxyphenylacetic acid, leading to immune system dysfunction and CD-related inflammatory responses [61]. Wikoff et al. [62] in a broad MS-based metabolomics study on plasma samples extracted from germ-free and conventional mice, revealed a significant effect of the microbiome on mammalian blood metabolites especially amino acid ones (like indole-containing metabolites derived from tryptophan). Other phenyl groups containing organic acid levels were also affected by the presence of gut microbes [62]. Shi et al. [63] using 16S rDNA and metabolomics sequencing, evaluated the changes in CD patients’ fecal microbial composition and metabolome characteristics. According to their results, CD patients showed an elevated abundance of *Streptococcus*, *Lactobacillus*, Veillonella, and *Allisonella* and a reduced abundance of *Ruminococcus*, *Faecalibacterium*, *Blautia*, *Gemmiger*, and *Anaerostipes*, indicating the imbalance of CD fecal microbiota. There were also differences in 222 fecal metabolites, mainly the levels of amino acids and their derivatives, between CD and HC groups showing the effects of CD on fecal-related terminal metabolites. A significant correlation was observed between the changes in gut microbiota and changes in fecal metabolite levels [63]. Girdhar et al. [64] in a recent study using the fecal samples of children who developed CD at the end of sampling (CD progressors) and healthy controls evaluated the role of gut microbiota and microbial metabolites in CD onset. Their results demonstrated that CD progressors had a distinct gut microbiota composition and 26 plasma metabolites, 5 cytokines, and 1 chemokine were significantly altered in them. Importantly, a microbiota-derived metabolite, taurodeoxycholic acid (TDCA), was elevated in CD progressors’ plasma, which proposed to have an enhancing effect on CD pathogenesis progression [64]. Previous studies on fecal samples of CD patients and HC showed that untreated and treated CD patients were significantly different from HC subjects in terms of short-chain fatty acids (SCFAs) such as butyrate, which are known as one of the important gut microbiota bio-products [65–67]. Di Cagno et al. [68] demonstrated a significant difference between the composition of the fecal microbiota of celiac children subjected to GFD and healthy controls mainly characterized by a lower abundance of *lactobacilli*, *enterococci*, and *bifidobacteria* in CD children. The analysis of fecal and urine metabolome by gas-chromatography mass spectrometry-solid-phase microextraction and 1 H-Nuclear Magnetic Resonance showed a marked change in volatile organic compounds and free amino acids in CD patients samples.

#### Malabsorption, energy metabolism and metabolite profile changes in CD

As stated before, gluten protein consumption leads to abnormal immune responses, which damages the small intestinal villi resulting in a reduction in the absorption surface area of the intestine resulting in nutrient malabsorption [69]. Accordingly, diarrhea, weight loss, nutritional deficiencies, and altered blood parameters are known as CD-related clinical characteristics that are especially present in subjects who do not have strict adherence to GFD treatment [70]. Although strict adherence to a GFD can improve nutritional status, it does not completely normalize nutritional deficiencies [71]. The changes in concentrations of methionine, choline, and choline-derived lipids were observed in CD patients’ samples, demonstrating an important effect of CD on one-carbon metabolism [38, 72–75]. Calcium malabsorption resulting in parathyroid hormone secretion alterations and changes in vitamin D metabolism causing mineral metabolism impairment are also observed in CD [76]. Upadhyay et al. [77] compared the metabolic profile of CD patients’ intestinal mucosa (relative to the disease controls), blood plasma, and urine (relative to the healthy controls) samples using NMR spectroscopy and multivariate analysis and observed the changes in Pro, arginine (Arg), glycine (Gly), histidine (His), glutamate (Glu), aspartate (Asp), tryptophan (Trp), fumarate, formate, succinate (Succ), glycerophosphocholine (GPC) and allantoin (Alln) in the small intestinal mucosa and changes in Pro, Arg, Gly, alanine, Glu, Gln, glucose (Glc), lactate (Lac), acetate (Ace), acetoacetate (AcAc), β-hydroxybutyrate (β-OHB), pyruvate (Pyr), Succ, citrate (Cit), choline (Cho), creatine (Cr), phosphocreatine (PCr) and creatinine in blood plasma and changes in Pro, Trp, β-OHB, Pyr, Succ, N-methylnicotinamide (NMN), aminohippurate (AHA), indoxyl sulfate (IS), and Alln in urine samples of CD patients [77]. Khalkhal et al. [69] evaluated serum metabolite levels of CeD patients relative to healthy controls using NMR spectroscopy and multivariate analysis and found that there is a distinct pattern in terms of metabolic signature in serum samples of celiac patients relative to the controls, which were related to lipid, carbohydrate, and amino acid metabolism. In a study conducted by Bertini et al. [38], differences in the metabolic profiles of CD patients’ plasma samples were observed in comparison with healthy controls; particularly glycolysis was reduced in untreated CD patients. Decreased lipids in sera and an increased level of ketone body 3-hydroxybutyric acid was also observed in CD patients’ specimens, which was supposed to be related to the increased beta-oxidation along with malabsorption. GFD adherence was reported to be effective in the normalization of the main energy metabolic pathway and recovery of villous functioning [38]. Actually, impairment of glycolysis causes a decrease in the level of pyruvate and lactate and an increase in the level of glucose in serum samples of CD patients [78]. As a result, lipid β-oxidation, as the second important metabolic pathway will be increased to produce energy by broking down the lipids. The lipid intake is also lower than a normal condition due to malabsorption and this phenomenon explains lower levels of lipids in sera followed by increased use of ketonic bodies as a more important source of energy in untreated CD patients [78].

### Lipidomic changes in CD

Lipids are essential metabolites that have key biological functions and play important roles in signaling, metabolism, and energy storage [79, 80]. The principal components of the cell membrane are lipids, which are affected by internal and external factors such as genetics, diet, lifestyle, inflammation, diseases, and drugs [81, 82]. Alterations in lipids profile and disturbances in their homeostasis can be associated with important diseases [83, 84]. In 2005, the international lipid classification and nomenclature committee on the initiative of the LIPID MAPS Consortium established a comprehensive classification system for lipids based on defined chemical and biochemical principles, and lipids have been classified into eight categories based on ketoacyl and isoprene groups: fatty acyl, glycerolipids, glycerophospholipids, sphingolipids, sterols, prenol lipids, saccharolipids, and polyketides. Diversity in the classification and structure of lipids has made their analysis a challenge [85, 86]. The total lipid content of a cell, tissue, or organism is called lipidome [87]. Lipidomics, which is a new discipline that emerged in 2003, is a subfield of metabolomics to study the biological lipidomes and lipid metabolism using the principle and techniques of analytical chemistry [26, 88]. In fact, lipidomics or lipid profiling studies evaluate the structure and function of lipids in a cell or organism, as well as their interactions with other cellular components and are important in defining lipid-related diseases underlying mechanisms [88, 89]. During the last decade, much attention has been paid to the use of lipidomics for better understanding the pathological mechanisms of CD and discovering its related novel biomarkers [90, 91]. As CD is characterized by increased intestinal permeability and dissemblance of its tight junctions and due to the importance of membrane lipid components in controlling barrier integrity, evaluating lipid profile in CD patients is of great importance [92, 93].

**Fig. 2:**
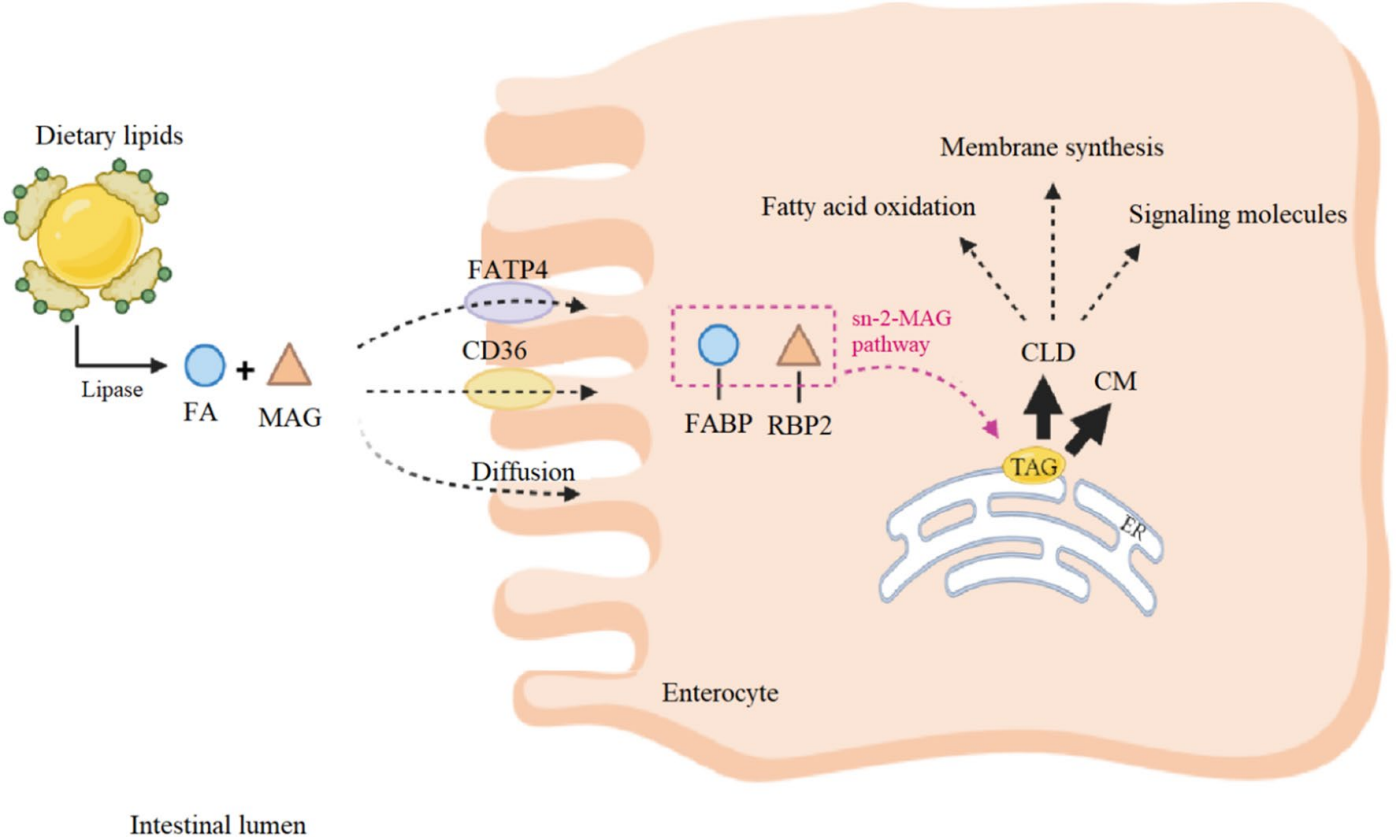
The metabolic fate of dietary lipids within enterocytes. *CLD* Cytoplasmic lipid droplet, *CM* chylomicron, *ER* endoplasmic reticulum, *FA* fatty acid, *FABP* fatty acid-binding protein, *FATP4* fatty acid transport protein 4, *MAG* monoacylglycerol, *RBP2* retinol-binding protein 2, *TAG* triacylglycerol

Actually, the absorption of food-derived fats and their distribution among peripheral tissues take place in the small intestine [94]. Food-derived lipids are hydrolyzed by lipases in the intestinal lumen and produce products like fatty acids (FAs) and monoacylglycerols (MAGs) [95]. FAs and MAGs cross the apical membrane of the enterocyte through the concentration gradient (passive diffusion) or via fatty acid transport protein 4 (FATP4) and CD36 [94, 95]. After entrance to the enterocytes, FAs and MAGs are bound by fatty acid-binding proteins (L-FABP and I-FABP) and retinol-binding protein 2 (RBP2), respectively, and utilized for triacylglycerol (triglyceride; TAG) re-synthesis in the endoplasmic reticulum (ER) via the sn-2-MAG pathway [94, 96]. The synthesized TAG will contribute to chylomicron (CM) or cytoplasmic lipid droplet (CLD) formation [96]. FAs and other lipids released from CLDs in enterocytes can contribute to membrane synthesis, fatty acid oxidation (FAO), or serve as signaling molecules [96, 97] (Fig. 2). These signaling roles might be important in controlling infection and inflammation [98]. CD-induced inflammation (through the production of eicosanoids) and malabsorption can cause changes in lipid profile of biological samples [99].

As carnitine is a vitamin-like compound that is involved in the beta-oxidation of long-chain fatty acids, which enters the blood circulation in the form of acylcarnitine, Bene et al. [100] compared the levels of free carnitine and acylcarnitine in the plasma samples of CD patients and healthy controls. All patients were under treatment with a GFD for at least one year. Their results showed that, although the plasma level of free carnitine did not differ between the two studied groups, a significant decrease in acylcarnitine levels was observed in CD patients. The levels of propionyl, butyryl, hexanoyl, octanoyl, octenoyl, decanoyl, cecenoyl, lauroyl, myristoyl, myristoleyl, and oleylcarnitine were also decreased in CD patients’ samples. They concluded that the metabolism of carnitine esters is affected by CD condition, and gluten withdrawal alone is not effective in normalizing all elements of the disturbed carnitine homeostasis [100]. Steel et al. [99] investigated whether the status of serum phospholipids can reflect the status of small intestinal mucosal fatty acids in CD patients. Pediatrics with active and under GFD CD as well as healthy individuals were included in this study. According to their findings, marginal differences in terms of serum phospholipids levels were observed between CD patients and controls. A significant difference was observed between the intestinal mucosal fatty acid composition of active CD patients and control subjects. Actually, the level of linoleic acid was decreased, while its derivatives were elevated. The level of Mead acid [(20:3(n-9)] was increased, with an increased ratio of Mead acid to arachidonic acid level, suggesting a deficiency in essential fatty acids. The level of these fatty acids during remission was not different from the control group. This study showed that the abnormality of intestinal mucosal fatty acids in CD patients was not reflected in their serum levels. It was also pointed out that the analysis of intestinal mucosal fatty acids is more accurate than serum phospholipids analysis [99]. Solakivi et al. [101] investigated the serum fatty acids profile of adults with CD at the time of diagnosis and after one-year treatment with a GFD in comparison to the healthy controls. In this study, an increase in palmitic acid, palmitoleic acid, stearic acid, and oleic acid was observed in CD patients compared to the control group and this increase persisted during remission. On the other hand, linoleic acid, alpha-linoleic acid, dihomo-gamma linoleic acid (DGLA), arachidonic acid (AA), eicosapentaenoic acid (EPA), docosapentanoic acid (DPA), and docosahexaenoic acid (DHA) were decreased in patients compared to controls. DPA and DHA increased significantly during remission, but their values were still lower than in the control group. During remission, the concentration of long-chain polyunsaturated fatty acids such as AA was still lower than in the control group, which indicated the insufficiency of essential fatty acids elongation and desaturation [101]. Sen et al. [102] applied lipidomics in a longitudinal study setting in children who progressed to CD at a mean age of 4.8 years (CD progressors) from the Type 1 Diabetes Prediction and Prevention (DIPP) cohort compared to controls. CD progressors had a different lipid profile than the control group and showed higher amounts of triacylglycerols (TGs) of low carbon number and double bonds and a decreased level of phosphatidylcholines by age 3 months. The differences were intensified with age but were not observed in umbilical cord blood [102]. Baldi et al. compared the serum FA profile of CD patients with healthy controls and observed a different composition of free circulating fatty acids (comprising SCFAs) in CD subjects, with a strong positive association between CD and butyric acid. They considered butyric acid to be a potential biomarker for CD screening [103]. Martín-Masot and his colleagues [104] tried to recognize the potential changes in the metabolic network of children with CD treated with a GFD in comparison to healthy control siblings. Their findings showed minor but significant alterations in the lipid metabolism of CD subjects with a particular affectation of steroids and derivatives, glycerophospholipids, glycerolipids, and fatty acyls. An increase in isobutyrate and 3-hydroxyisobutyric acid and a decrease in elaidic acid, linoleic acid, and stearic acid was also observed in serum samples of newly diagnosed CD patients by Khalkhal and her co-workers study [47].

CD is considered a complex multiorgan disease that patients may suffer sometime associated with major depressive disorder (MDD) besides gastrointestinal manifestations [105, 106]. Therefore, Van Hees et al. [107] investigated whether serum levels of docosahexanoic acid (DHA) and EPA as essential n-3 polyunsaturated fatty acids (PUFA), have a role in the association between CD and MDD, hypothesizing that GFD might be accompanied by lower DHA and EPA intake and serum levels leading to the higher risk of MDD in patients. Their study was conducted on under GFD treatment (more than 2 years) adult CD patients (with and without MDD) and healthy volunteers who did not use n-3 PUFA supplements. They showed that mean serum DHA levels were significantly higher in CD patients, but there was not any difference in serum EPA between them. Moreover, there was no relationship between serum levels of DHA and EPA and the state of depression, and the assumption that the use of n-3 PUFA supplements in CD may reduce the risk of MDD was rejected [107].

The composition of red blood cells (RBCs) membrane fatty acids reflects the interaction between dietary fatty acids intake and endocrine and immunological changes [108]. Riezzo et al. [109] performed a lipidomic evaluation of the fatty acid composition of the RBCs membrane of CD patients, at the time of diagnosis and following 1-year GFD adherence, in comparison to the healthy controls. The results showed the presence of pro-inflammatory FA profile in CD subjects and 1-year GFD adherence could not restore FA concentrations to normality. By observing an increased concentration of AA in CD patients on an unrestricted diet, they considered AA potential to be a putative marker of CD. They assumed this procedure, to be easier and non-invasive in comparison to the evaluation of the intestinal mucosal FA pattern for evaluating therapeutic interventions in CD patients by using FA [109].

### Metabolite and lipidomic profiles changes according to the interaction network

As there is a close connection between metabolites and genes, metabolites are sometimes called “canaries” of the genome [34]. Gene targeting at the RNA level has the potential to be considered as a therapeutic strategy for several metabolism errors induced disorders and promises to significantly advance current understandings of different disorders’ mechanisms of pathology [110]. As CD patients are at increased risk of metabolic syndrome and since many genes are involved in its pathogenesis, studying CD metabolites affecting genes is of great importance [7, 111].

**Fig. 3:**
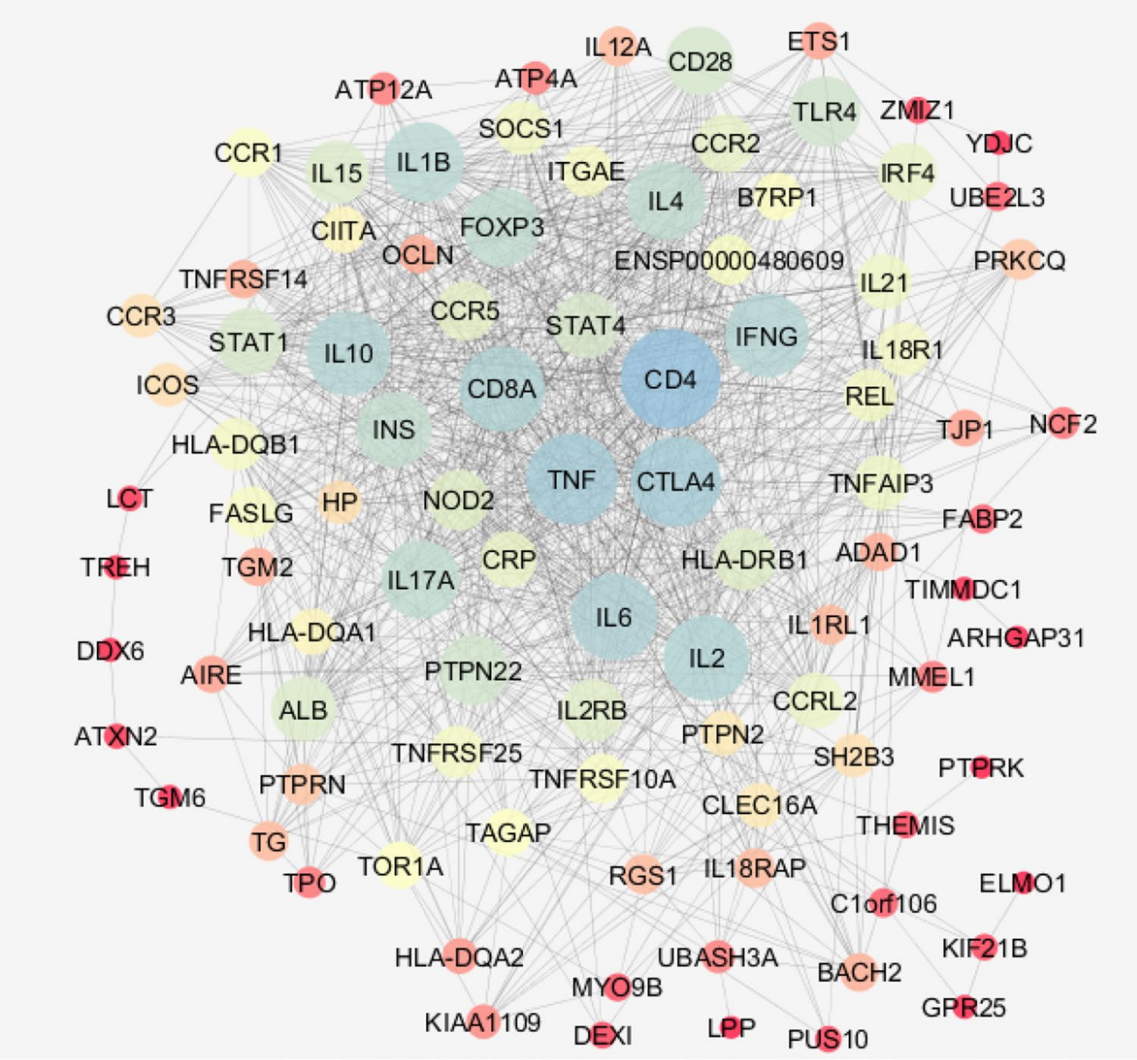
Protein–protein interaction network of CD-related genes

**Fig. 4:**
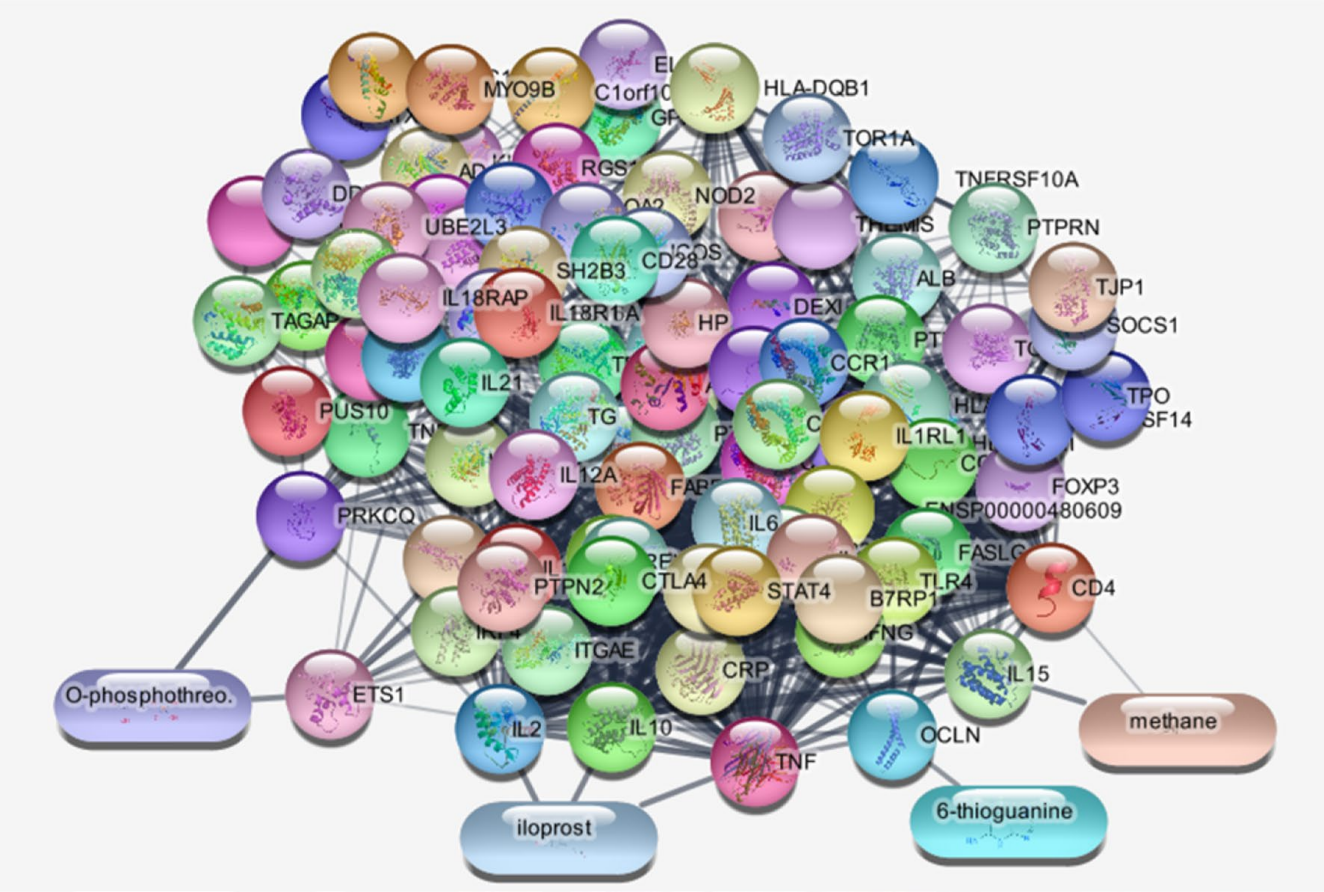
The metabolite-protein interaction network using STITCH

The 100 top CD-related genes were investigated from “disease query” of STRING database. The network was constructed via Cytoscape software version 3.7.2. The main connected component of the network was analyzed to find the hubs and bottlenecks (see Fig. 3). The 10 top nodes based on degree and betweenness were selected as the hubs and bottlenecks, respectively. The common hubs and bottlenecks were identified as hub-bottlenecks (see Table 1). As it is shown in Fig. 4 the metabolites related to the 100 investigated genes that were connected to the interactome were recognized via STITCH. Based on network analysis CD4, TNF, CTLA4, IL6, and IL2 are five key genes which play critical roles in CD development. CD4, TNF, and IL2 are the central genes which are connected to the introduced metabolites.

**Table 1:** The five crucial nodes related to CD

Display name	Degree	Betweenness centrality	Closeness centrality	Stress
CD4	63	0.075	0.676	7236
TNF	56	0.046	0.635	5418
CTLA4	55	0.054	0.639	4890
IL6	52	0.035	0.618	4284
IL2	51	0.041	0.614	3476

## Conclusion

Gluten induced intestinal mucosal damages disrupt the intestinal microbiota with a spectrum of simple dysbiosis and changing in metabolomic and lipidomic states with consequences on both physical and mental health of affected individuals. The present review indicates the importance of performing metabolomic- and lipidomic-based studies in CD patients with the aim of diagnostic and therapeutic biomarker discovery. Actually, metabolomics and lipidomics signatures could be considered the best strategies for a deeper investigation of CD pathogenesis. The published results, if confirmed in large prospective cohort studies, may also be useful in lipidomic-metabolomic-based risk assessment of subjects at higher risk of CD. Further studies, especially in pediatric populations, are needed to expand the current knowledge on this basis.
